# A Rapid One-Step Process for Fabrication of Biomimetic Superhydrophobic Surfaces by Pulse Electrodeposition

**DOI:** 10.3390/ma10111229

**Published:** 2017-10-25

**Authors:** Shuzhen Jiang, Zhongning Guo, Guixian Liu, Glenn Kwabena Gyimah, Xiaoying Li, Hanshan Dong

**Affiliations:** 1 School of Electromechanical Engineering, Guangdong University of Technology, Guangzhou 510006, China; piercejiang88@gmail.com (S.J.); znguo@gdut.edu.cn (Z.G.); lgx0904@163.com (G.L.); gk.gyimah@yahoo.com (G.K.G.); 2 School of Metallurgy and Materials, The University of Birmingham, Edgbaston, Birmingham B15 2TT, UK; x.li.1@bham.ac.uk

**Keywords:** biomimetic, superhydrophobic, contact angle, pulse electrodeposition, duty ratio

## Abstract

Inspired by some typical plants such as lotus leaves, superhydrophobic surfaces are commonly prepared by a combination of low surface energy materials and hierarchical micro/nano structures. In this work, superhydrophobic surfaces on copper substrates were prepared by a rapid, facile one-step pulse electrodepositing process, with different duty ratios in an electrolyte containing lanthanum chloride (LaCl_3_·6H_2_O), myristic acid (CH_3_(CH_2_)_12_COOH), and ethanol. The equivalent electrolytic time was only 10 min. The surface morphology, chemical composition and superhydrophobic property of the pulse electrodeposited surfaces were fully investigated with SEM, EDX, XRD, contact angle meter and time-lapse photographs of water droplets bouncing method. The results show that the as-prepared surfaces have micro/nano dual scale structures mainly consisting of La[CH_3_(CH_2_)_12_COO]_3_ crystals. The maximum water contact angle (WCA) is about 160.9°, and the corresponding sliding angle is about 5°. This method is time-saving and can be easily extended to other conductive materials, having a great potential for future applications.

## 1. Introduction

Surface wettability is one of the most important properties of solid materials [1,2]. Superhydrophobic surfaces with a water contact angle (WCA) greater than 150° and a sliding angle less than 10° have attracted considerable attention due to their unique behavior for both fundamental research and industrial application in the field of self-cleaning [3,4], anti-icing [5,6,7], anti-corrosion [8,9,10], water-oil separation [11], etc. The superhydrophobic phenomenon can be attributed to a rough surface with special micro/nano structures and/or surface chemical composition with low surface free energy.

Inspired by some particular natural creatures, such as lotus leaves [12,13], rose petal [14], rice leaves [15,16], and complex eyes of mosquito [17], a great number of processes were developed to fabricate the artificial superhydrophobic surfaces, including, but are not limited to, chemical etching [18,19,20], electrochemical etching [21,22], solution-immersion [9,23], sol-gel processing [24,25], laser processing [26,27], chemical vapor deposition [28], electrospinning [29], and hybrid processes [30,31,32,33]. However, most of the above mentioned methods involved some drawbacks, such as environmentally unfriendly chemical treatments, high vacuum and ultraclean working conditions, expensive equipment (e.g., photolithographic devices), or multi-step and time-consuming processing procedures, which have limited their wider industrial applications.

Because of its attractive combination of simplicity, cost-effectiveness, and high efficiency, direct current (DC) electrodeposition [8,10,34,35,36,37,38,39,40] has drawn great interest of researchers for the generation of superhydrophobic surfaces on conductive substrates. Moreover, large-scale fabrication regardless of the geometric shape of workpieces is a distinct advantage of cathodic electrodeposition in industrial applications. In addition, the surface morphology of the electrodeposited layer can be regulated by tuning the electrodeposition parameters, such as composition and concentration of the electrolyte, temperature, electrical parameters, and processing time. Due to their effect on improving the heat stabilization and corrosion resistance of advanced functional materials, rare earth elements (REE) were used in the fabrication of superhydrophobic surfaces [8,34,35].

When compared with conventional DC electrodeposition, pulse electrodeposition offers greater control over the structure and properties of electrodeposits by introducing some unique electrical parameters (such as frequency and duty ratio). They have considerable influence on the nucleation rate of crystals and the growth rate of crystalline grains during the process of electrocrystallization and finally determine the size and shape of crystalline grains. Within a period of pulse current, during the on-time, some cations near the cathode deposit on the surface continuously, while during the off-time, the discharging cations migrate from the bulk solution towards the vicinity of the cathodic electrode, thus leading to the effective recovery of the cation concentration of the dilute cathodic area. Meanwhile, the off-time intervals also restrain the evolvement of epitaxy and stop the further growth of crystalline grains [41,42]. Therefore, pulse electrodeposition has different forming mechanisms when compared with DC electrodeposition without off-time intervals. 

In addition, as compared with conventional DC electrodeposition, pulse electrodeposition has a higher peak current density [43,44]. Its duration and ratio of on-time and off-time can be adjusted, which enable pulse electrodeposition to be conducted under relatively short intervals and at relatively high current densities. Due to the high on-time current density, which can be several orders higher than the current density applied under the DC electrodeposition, pulse electrodeposition significantly increases the nucleation rate of crystals, thus increasing the deposition efficiency. When compared with DC electrodeposition, the pulse electrodeposition has such advantages as existence of a thin cathodic diffusion layer, quick supplement of adsorptive ions, high deposited efficiency, and convenience of surface morphology regulation.

However, to the best of our knowledge, only a few researches into the fabrication of superhydrophobic surfaces by pulse electrodeposition have been conducted and reported [38]. Notwithstanding the fact that these researches have revealed some advantages over the DC electrodeposition, no systematic work has been conducted to investigate the effect of the electrical parameters (such as duty ratio) of pulse current on the morphology of deposition layer, and their impact on the wettability performance.

Therefore, in this research, a rapid one-step process for the fabrication of superhydrophobic surfaces was developed using pulse electrodeposition with an REE containing electrolytic solution. The effect of duty ratio of pulse current on the surface morphology and wettability was specifically investigated. Based on the experimental results, the formation mechanism of the superhydrophobic surfaces during pulse electrodeposition is discussed. This method could be easily extended to other conductive materials and has great potential for industrial applications.

## 2. Materials and Methods

### 2.1. Materials and Sample Preparation

Anhydrous ethanol and myristic acid of analytical grade were purchased from Tianjin Damao Chemical Reagent Factory (Tianjin, China) and used as received. Lanthanum chloride (LaCl_3_·6H_2_O) was obtained from Tianjin Kemiou Chemical Reagent Co., Ltd. (Tianjin, China), and was used without further purification. Commercially pure copper sheets with a thickness of 1 mm were used as the substrate material for the deposition of superhydrophobic coatings. Copper plates with a size of 30 mm × 30 mm × 1 mm were abraded with silicon carbide papers (from 800 to 2000 grades), and then ultrasonically degreased in anhydrous ethanol for 10 min, followed by ultrasonic cleaning with deionized water for 10 min before being dried under atmospheric conditions.

### 2.2. Pulse Electrodeposition

**Figure 1:**
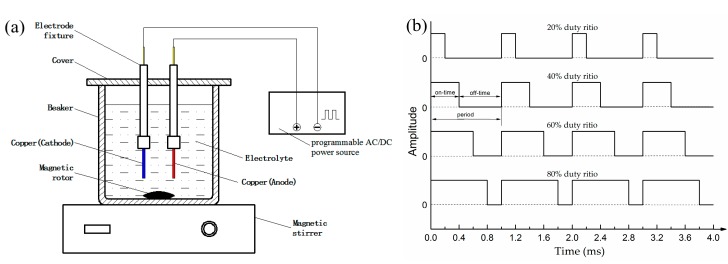
Schematic diagram of (**a**) a pulse electrodeposition setup and (**b**) waveforms of the pulse current applied in the experiments.

Electrolytic solution was prepared by adding 0.04 M lanthanum chloride and 0.1 M myristic acid into 100 mL ethanol under magnetically stirring at ambient temperature. Two copper plates were taken as the anode and cathode with a distance of 2 cm in an electrolyte cell. Pulse currents of 30 V and 1000 Hz with different duty ratios ranging from 20% to 80% were applied to the two electrodes by using a programmable AC/DC power source (ELGAR SW5250A, AMETEK Programmable Power, San Diego, CA, USA). Here, in pulse current, duty ratio means the ratio of the on-time (T_on_) to the total period (T) of the waveform and can be expressed as a ratio or as a percentage. The composition of the electrolytic solution schematically shown in Figure 1b. In order to avoid contamination and mitigate the effects and the electrical parameters of the processes are summarized in Table 1, while a schematic diagram of the experimental setup is shown in Figure 1a and the waveforms of the pulse current applied in the experiments are of the reagent concentration, the used electrolyte was replaced by a new solution after fabricating every sample. After 10 min equivalent electrolytic time at room temperature under stirring condition (200 rpm) by using a magnetic stirring apparatus, the cathodic electrode was rinsed thoroughly several times with ethanol and distilled water and then was dried in an air condition. Subsequently, a cathodic surface with hierarchical micro/nano structure was obtained.

### 2.3. Sample Characterization

The surface morphology of the as-deposited surfaces was characterized by scanning electron microscopy (SEM, JEOL JSM 6060LV, Tokyo, Japan) at 10 kV. Surface roughness was measured by a surface profilometer (Ambios Technology XP-200, Santa Cruz, CA, USA). The average of six measurements at different locations on the samples were reported. The corresponding chemical composition was examined by Fourier transform infrared spectrophotometer (FTIR, Thermo Fisher Scientific NICOLET 8700, Waltham, MA, USA) and energy dispersive spectrometer (EDX, EDAX Genesis 60, Mahwah, NJ, USA). The crystallography information was determined by an X-ray diffractometer (XRD, Bruker D8, Billerica, MA, USA) with monochromatic Cu K*α* radiation (*λ* = 0.15418 nm), which was operated at 40 kV and 40 mA. Surface wettability was measured by a contact angle measurement instrument (XG-CAMB1, Shanghai Xuanyichuangxi Industrial Equipment, Shanghai, China) at ambient temperature for each surface. Water droplets with about 4 μL were dropped on the deposited layers from a distance of 0.2 cm by vibrating the burette. The sliding angles were measured with the assistance of a rotatable platform with an angle scale. The reported data were obtained as the average of five measurements at different locations on the samples. The bouncing phenomenon was captured by a high speed camera (Fastec HiSpec5, San Diego, CA, USA) with 400 fps.

## 3. Results

### 3.1. Surface Morphology

**Figure 2:**
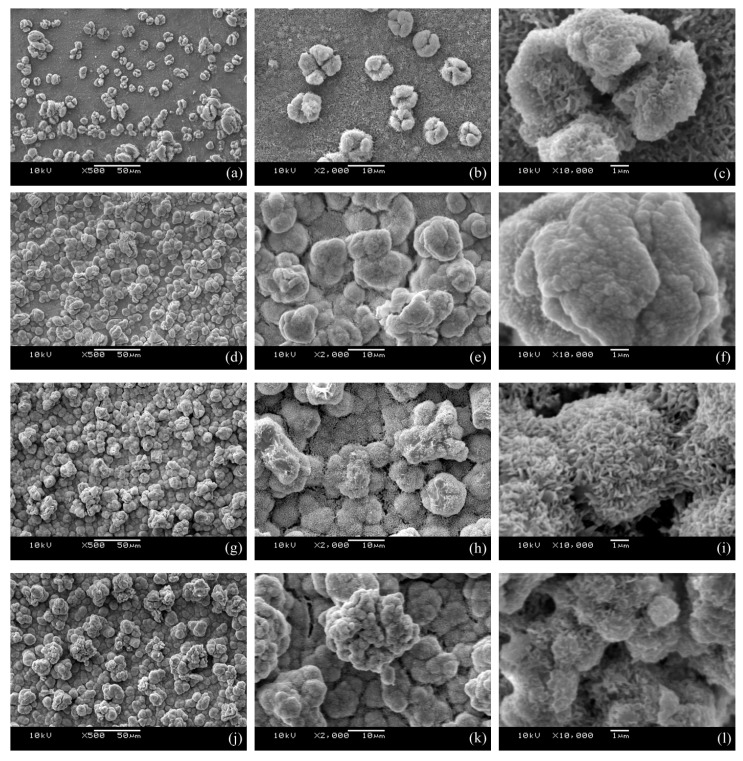
Scanning electron microscopy (SEM) images of the as-prepared surfaces deposited at 30 V and 1000 Hz pulse current for different duty ratio. (**a**–**c**) 20%; (**d**–**f**) 40%; (**g**–**i**) 60%; (**j**–**l**) 80%.

Surface morphology plays an important role in determining the wetting properties of a surface. Figure 2 shows the SEM images of the as-prepared layers deposited at 30 V and 1000 Hz for various duty ratios after 10 min equivalent electrolytic time. Figure 2a–c show the morphology of the cathodic surface obtained under 20% duty ratio condition. As shown in Figure 2a, some micro particles with a diameter around 5–20 μm are distributed unevenly and sparsely on the relatively smooth and flat surface with nano-scale roughness. Almost all of the micro particles showed cracks on the surface, some of which are similar to coffee beans; some even had cross cracks that make them look like a four-leaf clover (see Figure 2c). When the duty ratio was extended to 40%, the amount of the three-dimensional (3D) crystallites is considerably increased although their size is only slightly increased (Figure 2d–f) as compared with the surfaces deposited using 20% duty ratio (Figure 2a–c). As evidenced in Figure 2d, the crystallites covered nearly the whole surface, and some crystallites even stacked together to form local clusters that led to heterogeneous surface structures. A local magnified image (Figure 2f) showed the papilla-like micro structure with nano scale asperities on the as-prepared surface.

The morphology of the pulse electrodeposited surfaces under a prolonged duty ratio of 60% is shown in Figure 2g–i. The density and size of the particles continuously increased, which made them cluster around and cover the surface completely. Furthermore, interconnected hair-like nano structure with a dimension of about 1 μm long and 50 nm wide was observed on the micro-scaled asperity structures. The image at a high magnification, as shown in Figure 2i, demonstrates that the combination of micro-nano dual scaled structure resembles the surface characteristics of lotus leaf. Thus, the surface obtained under this condition exhibited a highly textured morphology with a hierarchical micro-nano structure. With a further increasing duty ration to 80%, the SEM images shown in Figure 2j–l clearly reveal that the particles still grew and formed the larger clusters with an anisotropic crystal growth, which makes the number of clusters correspondingly decrease. As a whole, with the increase of the duty ratio, the size of the particles or clusters was enlarged constantly while the density of them increased first and then decreased.

**Figure 3:**
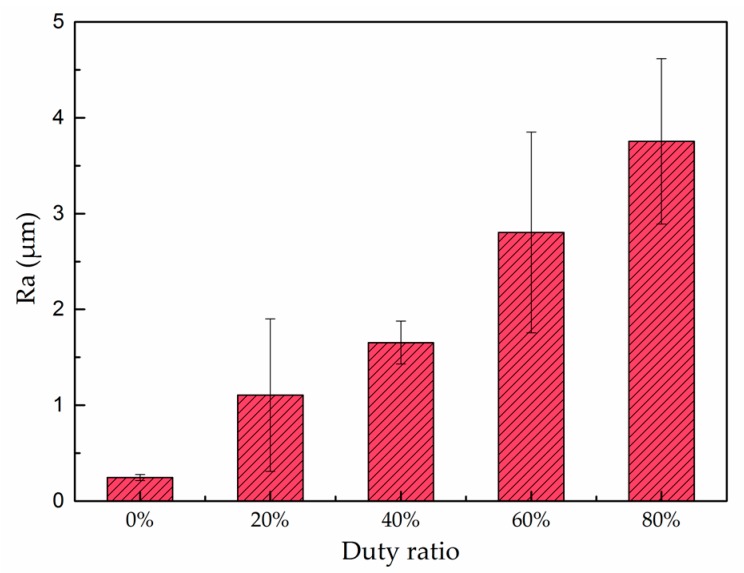
The surface roughness of the polished copper plate and the as-coated surfaces deposited at 30 V and 1000 Hz pulse current for different duty ratios.

As shown in Figure 3, the Ra of bare coppers after polishing is only about 0.25 μm while the Ra of the as-prepared surfaces coated under 20%, 40%, 60%, and 80% duty ratio is 1.11, 1.65, 2.80, and 3.75 μm, respectively. Clearly, the Ra value increased with the duty ratio used for the deposition. 

### 3.2. Phase and Chemical Composition

**Figure 4:**
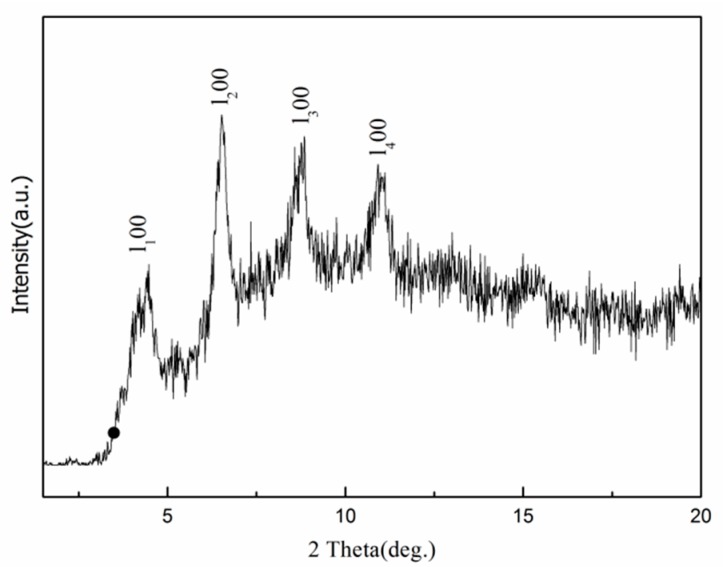
X-ray diffractometer (XRD) pattern of the superhydrophobic surface generated by pulse electrodeposition under 60% duty ratio.

In this study, XRD, FTIR, and EDX spectra were employed to analyze the phase and chemical composition of the as-prepared surfaces. Recorded in the 2*θ* range of 1.5°~20.0°, the XRD pattern of the superhydrophobic surface obtained using pulse current at 30 V, 1000 Hz, and 60% duty ratio is shown in Figure 4. A set of well-defined diffraction peaks marked with (l_n_,0,0) can be observed within the small angle region from 4.5° to 12.0°, which indicates that the as-prepared surface is crystallized and has a layer structure [45].

**Figure 5:**
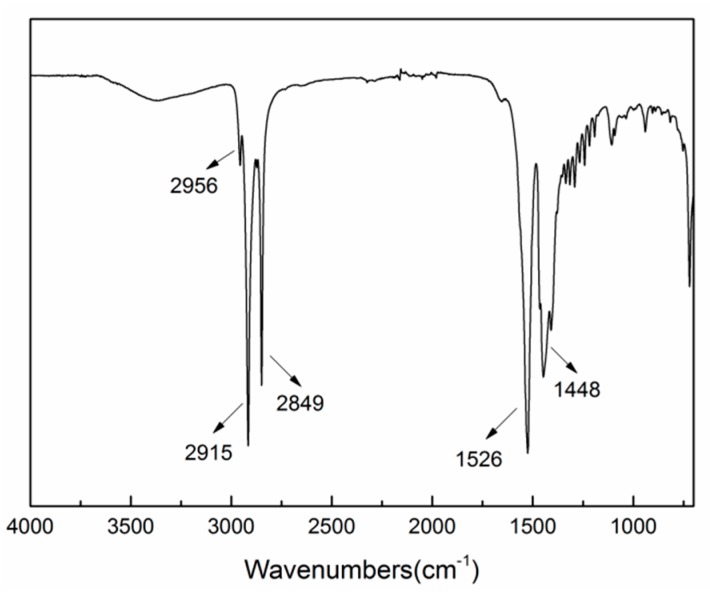
Fourier transform infrared spectrophotometer (FTIR) spectrum of the superhydrophobic surface obtained by pulse electrodeposition under 60% duty ratio.

In order to acquire the functional group information of the deposition layer, FTIR spectrum, as exemplified in Figure 5, was obtained from the as-prepared surface under 60% duty ratio. In the low frequency region, the corresponding absorption peak of free carboxyl group (–COO–) from myristic acid appears at 1701 cm^−1^ [19]. The deposition layer exhibited the adsorption peaks at 1526 cm^−1^ and 1448 cm^−1^, which may stem from asymmetric and symmetric stretches of carboxyl group. In the high frequency region, the absorption peaks at 2849 cm^−1^ and 2915 cm^−1^ can be ascribed to methylene groups(–CH_2_–) asymmetric and symmetric stretching vibrations, while the absorption peak at 2956 cm^−1^ are attributed to methyl groups(–CH_3_) asymmetric stretching vibrations. The surface energy of the –CH_3_ and the –CH_2_– groups are 24 mJ/m^2^ and 31 mJ/m^2^, respectively [46], which implies that the deposition layers have low free energy.

**Figure 6:**
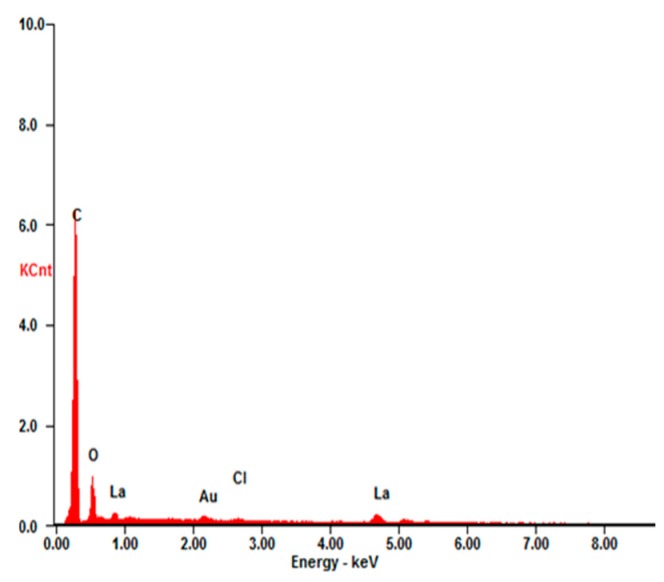
Energy dispersive spectrometer (EDX) spectrum of the superhydrophobic surface obtained by pulse electrodeposition under 60% duty ratio.

Figure 6 depicts a typical EDX spectrum from the as-deposited surface under 60% duty ratio and elements La, C, O, and Cl can be identified. As listed in Table 2, the atomic percentage of La/C/O is about 1:33.8:5.05. This is reasonably close to the La/C/O ratio (1:42:6) in La[CH_3_(CH_2_)_12_COO]_3_ if the experimental errors of EDX measurement of light elements (C and O) from rough surface are taken into account. Hence, it can be inferred that a low surface free energy material named lanthanum myristate (La[CH_3_(CH_2_)_12_COO]_3_) is deposited on the copper substrates.

### 3.3. Surface Wettability

**Figure 7:**
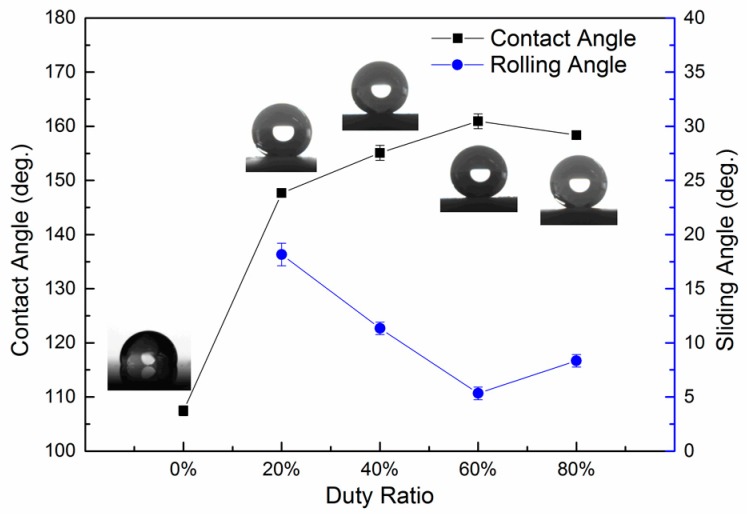
Water contact angle and sliding angle of pulse electrodeposited surfaces as a function of pulse current duty ratio.

In order to correlate the relationship between the duty ratio of the pulse current used and the wettability of the pulse electrodeposited surface, the contact angles of the as-prepared surfaces were measured and the results are shown in Figure 7. The contact angle increased from 107.5° for the polished copper modified with myristic acid (as shown in Figure 7 with 0% abscissa) to 147.7° with a sliding angle of about 18° when pulse electrodeposited at 30 V, 1000 Hz, and 20% duty ratio. When the duty ratio prolonged to 40%, the contact angle increased to 155.1° and the sliding angle reduced to 11°, indicating that the surface wettability almost reached the superhydrophobic state. As the duty ratio was further increased to 60%, the contact angle increased to 160.9° and the sliding angle further reduced to only 5°. However, further increasing the duty ratio to 80%, the contact angle decreased slightly to 158.7° and the sliding angle slightly increased to 8°, but still remains in the superhydrophobic state. More reproducible results of surface wettability could be obtained by following the protocol presented by Drelich, etc. [47,48].

**Figure 8:**
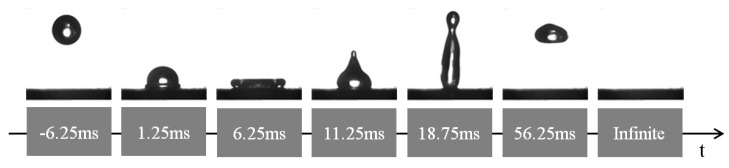
Time-lapse photographs of water droplets bouncing on the as-prepared surface at 60% pulse duty ratio.

If a surface is water repellent, water droplets tend to bounce instead of wetting the surface [49,50]. To verify the impact of hierarchical micro-nano structure on the long-term superhydrophobicity of the pulse electrodeposited surfaces, high-speed photography was employed to capture the bouncing phenomenon of the cathodic surface obtained under 60% duty ratio condition after exposure in the air for eight months. A water droplet fell freely from a height of 4 cm and hit the as-prepared surface with an impact velocity of about 88.5 cm·s^−1^. It can be observed from Figure 8 that the water droplet completely left the surface without leaving any trace or contamination on the surface. However, it should be noticed that at 18.75 ms, the droplet almost separated into two, due to the slightly increased adhesion of the water on the surface. This state has changed slightly after exposure in the air in a period of eight months. Despite of this phenomenon, the as-prepared surface still exhibited excellent non-sticking properties. This indicates that the surface was superhydrophobic with a long-term stability and was similar to the lotus effect with significant self-cleaning property.

## 4. Discussion

### 4.1. The Superhydrophobicity of the Pulse Electrodeposited Surfaces

As has been shown in Figure 2, the surface morphology of the pulse electrodeposited surfaces is a function of the pulse current duty ratio. When compared with the un-coated Cu substrate, all of the pulse electrodeposited surfaces showed significantly increased water contact angle or wettability; the water contact angle of the pulse electrodeposited surfaces increased from 147.7° to 160.9° when increasing the pulse current duty ratio from 20% to 60%, respectively. This might be attributed to the formation of low surface free energy lanthanum myristate (La[CH_3_(CH_2_)_12_COO]_3_) formed during the pulse electrodeposition process (Figure 3 and Figure 4) in view of the gradually increased coverage of the substrate by the surface particles, as shown in Figure 2a through 2d to 2g. However, further increasing the duty ratio to 80% led to slightly reduced (158.7°), rather than increased, water contact angle, although both the surfaces are indeed fully covered. Clearly, this cannot be explained from a surface energy point-of-view and there should be other mechanisms in operation. 

Close examination of the morphology of the surfaces electrodeposited by 60% and 80% duty ratios has revealed a highly-textured surface with a hierarchical micro-nano dual scale structure. As evidenced in Figure 2i, interconnected hair-like nano-structures are superimposed on the surface of the micro-structures, which is similar to the characteristics of lotus leaf and could have contributed to the observed the superhydrophobicity with the highest water contact angles around 160° and the lowest sliding angles around 5°. However, the nano-structures formed on the 80% duty ratio deposited surface (Figure 2l) is not so developed as that formed on the 60% duty ratio deposited one (Figure 2i).

Based on the above observation, the superhydrophobicity of the pulse electrodeposited surfaces can be mainly ascribed as Cassie–Baxter state [51], describing by the equation as follows:

(1) $$\cos\theta_{r}=f_{1}\cos\theta -f_{2}$$

where $f_{1}$ and $f_{2}$ are the area fraction of the solid and air on the surface, respectively, and$\;f_{1}+f_{2}=1$; θ and $\theta_{r}$ are the intrinsic contact angle of the flat solid surface and apparent contact angle of the rough surface. In this study, the contact angles of the polished copper modified with myristic acid and the as-prepared surface with hierarchical micro/nano structure are about 107.5° and 160.9°, respectively. Input these data into the Equation (1), the value of $f_{1}$ and $f_{2}$ can be calculated as 0.0787 and 0.9213, respectively. This means that about 92.13% of the water droplet bottom is supported by the air cushion and only 7.87% water droplet bottom is in direct contact with the solid surface. The trapped air increases the interface between the water and the air, thus preventing the penetration of water droplets into the surface. This dual scale structure makes the water droplet suspend on the air cushion, resulting in a higher surface contact angle. This clearly indicates that the air cushion plays an important role in promoting the surface superhydrophobicity and it is the dual micro-& nano-structure formed during the pulse electrodepostion process with an optimal pulse current duty ratio that have contributed to the observed superhydrophobicity.

### 4.2. Effect of Pulse Current Duty Ratio on Surface Morphology

It is well known that electrocrystallization mainly includes three basic steps: generation of seed crystals, formation of crystal nuclei, and the growth of crystals. First, seed crystals emerge on the interface of the cathodic electrode and electrolyte. Then, the seed crystals continuously grow, relying on the gathering of the adsorptive ions on the interface, and form into crystal nuclei with critical size. Finally, the nucleus evolve into crystals. The size of crystalline grains is closely related to nucleation rate of crystals and growth rate of crystalline grains during the process of electrocrystallization.

In this study, the frequency of the pulse current is 1000 Hz, which means that the period of the pulse current is only 1 ms. During the on-time of the pulse current, some La^3+^ cations near the cathode reacted with myristic acid, leading to the formation of lanthanum myristate and some H^+^ irons on the cathodic surface at the same time. The free H^+^ ions received the electrons from the anode via the cell circuit and were reduced to hydrogen, gathering around the cathodic surface. According to the electrochemical mechanism, reactants and the data of surface chemical composition obtained through XRD and EDX, the reaction equations can be described as Equations (2) and (3).

(2) $${\mathrm{La}}^{3+}+3{\mathrm{CH}}_{3}{\left({\mathrm{CH}}_{2}\right)}_{12}\mathrm{COOH}\to \mathrm{La}{\left[{\mathrm{CH}}_{3}{\left({\mathrm{CH}}_{2}\right)}_{12}\mathrm{COO}\right]}_{3}+3H^{+}$$

(3) $$2H^{+}+2e^{-}\to H_{2}↑$$

The generation of H_2_ bubbles stirred the electrolyte and promoted the formation of the micro/nano structure on the cathodic surface. While during the off-time of the pulse current, the discharging cations migrate from the bulk solution towards the vicinity of the cathodic electrode, leading to the effective recovery of the cation concentration of the dilute cathodic area. Meanwhile, the off-time intervals also restrain the evolvement of epitaxy and stop the further growth of crystalline grains.

It thus follows from the above discussion that the duty ratios will affect the forming mechanism of electrodeposits by determining the nucleation rate of crystals and the growth rate of crystalline grains. Clearly, the duty ratios will play an important role in determining the surface morphology and hence the performance of superhydrophobicity. For example, when the duty ratio is only 20%, the on-time is 0.2 ms while the off-time is 0.8 ms, nucleation plays a key role rather than crystal growth, leading to the surface morphology with some micro particles distributed sparsely on the relatively smooth surface, as shown in Figure 2a. When the duty ratio is increased to 40%, the on-time increased and the proportion of crystal growth enhanced correspondently. The particles formed under such conditions have increased size and coverage as shown in Figure 2d. 

Further increasing the duty ratio to 60% increased the coverage of the particles with no further increase in their size. It is of great interest to find that interconnected hair-like nano-structures are superimposed on the surface of the micro-structures. Notwithstanding the fact that the mechanism involved is still under investigation, this duel micro-nano structure is similar to the characteristics of lotus leaf and could have contributed the observed superhydrophobicity with the highest water contact angles around 160°.

When increasing the duty ratio to 80%, which means that the on-time is 0.8 ms while the off-time is only 0.2 ms, the competitive crystal growth becomes more pronounced than nucleation, leading to the largest particle size when compared with the other surfaces obtained by a lower duty ratio. Indeed, when the duty ratio is too large, pulse electrodeposition will lose the advantage of having a thin cathodic diffusion layer and a quick supplement of adsorptive ions, thus approaching the effect of direct current electrodeposition. 

It can be observed from Figure 3 that the surface roughness of the as-deposited surfaces increased with the increase of the duty ratio employed for the pulse electrodeposition process. In general, the wettability of a surface increases with its surface roughness if the surface chemistry and surface energy remain constant. However, it is of interest to note from Figure 7 that the corresponding static contact angle firstly increased and then slightly decreased; the sliding angle firstly decreased and then slightly increased. This indicates that the as-prepared surface obtained under 60% duty ratio possessed the maximum static contact angle and the minimum sliding angle, although its surface roughness Ra (2.80 μm) is lower than the one (3.75 μm) obtained under 80% duty ratio. This seemingly abnormal result could be explained by the special dual micro-nano hierarchical structure that formed on the surface obtained under 60% duty ratio, which did not exist on the other as-prepared samples. As discussed above, the interconnected hair-like nano-structure (Figure 2i) has contributed to the superhydrophobicity. However, the existence of such nano-structures would not affect the Ra value measured by the profilometer used in this work. Thus, it follows that the dual micro-nano hierarchical structure actually should have greatly contributed to the best hydrophobicity performance [12].

In short, there is a close relationship between the superhydrophobic performance of the as-prepared surfaces and the duty ratio used to produce the surfaces. The duty ratios have affected the forming mechanism of electrodeposits by determining the nucleation rate of crystals and the growth rate of crystalline grains. 

## 5. Conclusions

In conclusion, a rapid one-step process was successfully developed to fabricate the biomimetic superhydrophobic surface by pulse electrodeposition with an electrolyte solution containing lanthanum chloride, myristic acid, and ethanol. The experimental results show that the duty ratio of the pulse current plays an important role in determining the surface morphology of the deposition layers. It is the combination of a dual micro-nano surface structure and a surface layer of La(CH_3_(CH_2_)_12_COO)_3_, with a low surface energy that has contributed to the superhydrophobicity with a contact angle as high as 160.9° and the corresponding sliding angle is about 5°. The excellent durability of the optimal cathodic deposition layer has been confirmed by the droplet bouncing tests after a period of eight months following the deposition. It is expected that this method could be easily extended to various conductive materials for the fabrication of functional surfaces with superhydrophobic properties. In the future research, the effect of other parameters of the pulse current will be studied.

## Figures and Tables

**Table 1:** Electrolytic and electrical parameters.

Electrolytic Composition	Value	Electrical Parameters	Value
LaCl_3_·6H_2_O (M)	0.04	Voltage (V)	30
CH_3_(CH_2_)_12_COOH (M)	0.1	Frequency (Hz)	1000
C_2_H_5_OH (mL)	150	Duty ratio	20%, 40%, 60%, 80%

**Table 2:** Composition of the as-prepared superhydrophobic surface obtained by EDX.

Element	Wt %	At %
C-K	63.73	84.16
O-K	12.74	12.62
Cl-K	1.61	0.72
La-L	21.92	2.50
Total	100	100
